# Bacterial Burden Declines But Neutrophil Infiltration and Ocular Tissue Damage Persist in Experimental *Staphylococcus epidermidis* Endophthalmitis

**DOI:** 10.3389/fcimb.2021.780648

**Published:** 2021-11-17

**Authors:** Susmita Das, Sukhvinder Singh, Ashok Kumar

**Affiliations:** ^1^ Department of Ophthalmology, Visual and Anatomical Sciences/Kresge Eye Institute, Wayne State University School of Medicine, Detroit, MI, United States; ^2^ Department of Biochemistry, Microbiology, and Immunology, Wayne State University School of Medicine, Detroit, MI, United States

**Keywords:** eye, retina, endophthalmitis, *Staphylococcus epidermidis*, innate immunity, inflammation

## Abstract

Coagulase-negative staphylococci (CoNS), including *Staphylococcus (S) epidermidis*, are responsible for ~70% of all post-surgical endophthalmitis, a potentially blinding eye infection. However, the pathobiology of CoNS endophthalmitis is limited to epidemiological and clinical case studies with few experimental studies. Here, we report both *in vitro* and *in vivo* models to study the pathobiology of *S. epidermidis *endophthalmitis in mice. We found that *S. epidermidis *is rapidly cleared from mouse eyes, and a relatively higher dose (i.e., 10^7^ CFU/eye) was needed to cause endophthalmitis. Our time-course study revealed that bacterial load peaked at 24 h post-infection followed by a gradual decline up to 72 h. A similar time-dependent decrease in levels of inflammatory mediators and Toll-like receptor (TLR) expression was also observed. In contrast, neutrophil infiltration continued to increase up to 72 h coinciding with significant retinal tissue damage and loss of visual function. *In vitro*, *S. epidermidis* induced the activation of various inflammatory signaling pathways (i.e., NF-kB, ERK, and P38) and the production of both cytokines and chemokines in mouse BMDMs, human RPE, and retinal Muller glia. Altogether, we show that bacterial burden is reduced in *S. epidermidis* endophthalmitis, while tissue damage and visual function loss continue. Thus, our study provides new insights into the pathogenesis of CoNS endophthalmitis.

## Introduction

*Staphylococcus (S) epidermidis* is a Gram-positive bacteria, which commonly present on the skin as a part of its normal flora (Otto, 2009; Brown and Horswill, 2020). However, along with other coagulase-negative staphylococci (CoNS), it remains a leading cause of nosocomial infections, in part, due to its inherent ability to acquire antibiotic resistance (May et al., 2014; Lee et al., 2018). Earlier considered to be an innocuous microbe, *S. epidermidis* is now studied as an opportunistic pathogen and the cause behind multiple diseases, including life-threatening antibiotic-resistant infections (Otto, 2009; Cui et al., 2019). In the eye, *S. epidermidis* and other coagulase-negative staphylococci (CoNS) have been reported to cause conjunctivitis, blepharitis, corneal ulcers, and endophthalmitis (Flores-Páez et al., 2015). In addition, CoNS are frequently recovered from aqueous and vitreous samples in postsurgical complications (Johnson et al., 1997; Priya et al., 2014; Teweldemedhin et al., 2017; Romanowski et al., 2021). One of the key virulence properties of *S. epidermidis* in causing ocular infections is attributed to its ability to create biofilms on intraocular and soft contact lenses (Faghri and Razavi, 2009; Konduri et al., 2021) as well as ocular prostheses (Moreno et al., 2020).

The severity of eye infections due to CoNS increases when the bacteria gain access to intraocular tissue, such as during endophthalmitis, a dreaded complication arising from post-operative or traumatic injuries, which can lead to vision loss (Giese and Mondino, 2001; Miller et al., 2019). The most common form of endophthalmitis is exogenous, which occurs when microbial organisms on/around the ocular surface, or from any external source, get inside the eye (Miller et al., 2019). However, microbes can enter the eye *via* hematogenous spread resulting in endogenous endophthalmitis (Gregory et al., 2007). Several bacterial and fungal pathogens have been reported to cause endophthalmitis; *Staphylococcus* species, particularly CoNS, such as *S. epidermidis*, accounts for almost 70% of all exogenous bacterial endophthalmitis (Smith et al., 1986; Kumar et al., 2013; Miller et al., 2019). On the skin, *S. epidermidis* not only is considered harmless but also exerts beneficiary roles in protecting the skin from infections by boosting innate immunity and outcompeting pathogenic bacteria (Naik et al., 2015; Nguyen et al., 2017). Similarly, *S. epidermidis* colonizing the healthy conjunctiva have been reported to have distinctive genotypic and phenotypic characteristics as compared to those causing ocular infections (Flores-Páez et al., 2015). However, *S. epidermidis* dwelling on conjunctiva and skin surrounding the eye can easily contaminate the medical devices used during ocular surgeries, allowing entry inside the eye (O'Day et al., 1982; Bode et al., 1985; Uçkay et al., 2009).

Despite the higher incidence of endophthalmitis due to *S. epidermidis* and other CoNS (Callegan et al., 2002; Lalitha et al., 2017; Malmin et al., 2021), our current understanding of the pathobiology and host–pathogen interactions during *S. epidermidis* endophthalmitis is limited to a few previous studies in rat and rabbit models (Scorza and Berni, 1966; Pleyer et al., 1992; Ravindranath et al., 1997; Oguz et al., 2004; Kim and Kim, 2011). Moreover, most of these experimental models were utilized to test the therapeutic efficacy of antibiotics. Studies are lacking to examine the disease process, i.e., the initiation, progression, and eventual termination of the host’s innate immune responses. This prompted us to carry out the current study with two main objectives: 1) to develop a mouse model of *S. epidermidis* endophthalmitis and 2) to study the interaction of *S. epidermidis* with innate immune and retinal residential cells.

## Results

### *S. epidermidis* Induces Endophthalmitis in B6 Mice at a Higher Infectious Dose

**Figure 1:**
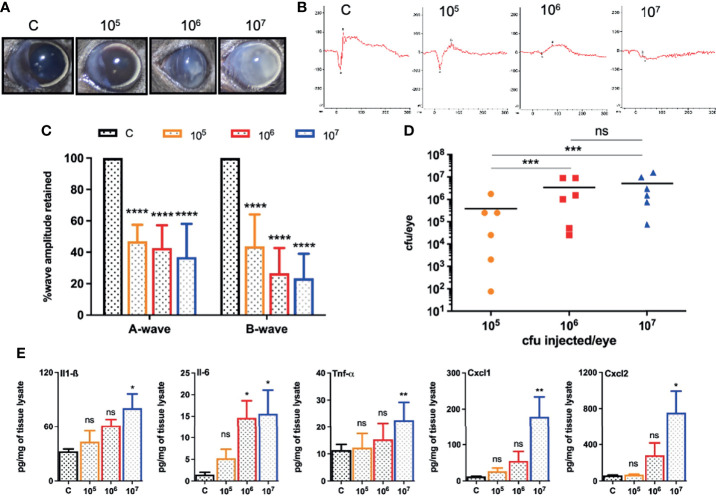
Intravitreal inoculation of *S. epidermidis* causes endophthalmitis in C57BL/6 mice. C57BL/6 mice eyes (n = 6 eyes each condition) were intravitreally injected with indicated colony forming units (cfu/eye) of *S. epidermidis* or PBS (control, C), and eyes were processed 24 h post-infection (p.i.). **(A)** Slit-lamp examination was performed, and photomicrographs were taken from representative eyes showing corneal haze/opacity. **(B)** Scotopic electroretinogram (ERG) analysis was performed to assess retinal function. **(C)** Bar graph showing percent a- and b-wave amplitude retained with respect to control eyes set at 100%. **(D)** At 24 h p.i. eyes were enucleated and homogenized, and the bacterial burden was estimated *via* serial dilution plating. **(E)** The lysates from infected and control eyes were subjected to ELISA to quantify protein levels of indicated inflammatory mediators. Statistical analysis was performed using ANOVA with Dunnett’s multiple comparison test **(C, E)** or Tukey’s multiple comparison test **(D)**, (*) p<0.05 (**) p<0.01 (***) p<0.001 (****) p<0.0001; ns, nonsignificant.

Because different pathogens cause experimental endophthalmitis at certain infectious doses (Gupta et al., 2019; Francis et al., 2020; Kumar et al., 2020; Livingston et al., 2021), we performed a dose-response study beginning with an intravitreal injection of 5,000 cfu of *S. epidermidis*, a dose required for *S. aureus* endophthalmitis (Singh et al., 2021). However, this inoculum was rapidly cleared from the mouse eyes resulting in transient inflammation (data not shown). Afterward, eyes were challenged with three higher doses (10^5^, 10^6^, and 10^7^ cfu) of *S. epidermidis*. Our data showed a dose-dependent increase in corneal haze, anterior chamber opacity, and hypopyon formation at 24 h post-infection (**Figure 1A**). The ERG analysis revealed that all *S. epidermidis* infected mouse eyes have reduced amplitudes of *a* and *b* waves, indicating significant loss of retinal function in comparison to uninfected control eyes (**Figures 1B, C** **)**. Eye challenged with 10^7^ cfu retained the least ERG response with flat wavefront. As expected, PBS injected control eyes did not show any bacteria growth, whereas the intraocular bacterial burden correlated with an infectious dose, i.e., the higher the injected inoculum, the more bacteria recovered from the eye (**Figure 1D**). Interestingly, we noticed that out of six eyes, three eyes in 10^5^ cfu, two eyes in 10^6^ cfu, and one eye in 10^7^ cfu groups had lower bacterial burden than the injected inoculum. The assessment of inflammatory mediators showed that *S. epidermidis* induced the production of inflammatory cytokines (IL1-β, IL-6, TNF-α) and chemokines (CXCL-1 and CXCL-2) in mouse eyes (**Figure 1E**). However, significant elevation was detected in eyes infected with 10^7^ cfu followed by 10^6^ cfu but not by 10^5^ cfu. These results indicate that *S. epidermidis* dose is a key determinant in the pathogenesis of endophthalmitis.

### *S. epidermidis* Infected Eyes Exhibit Pathology and Impaired Retinal Function

**Figure 2:**
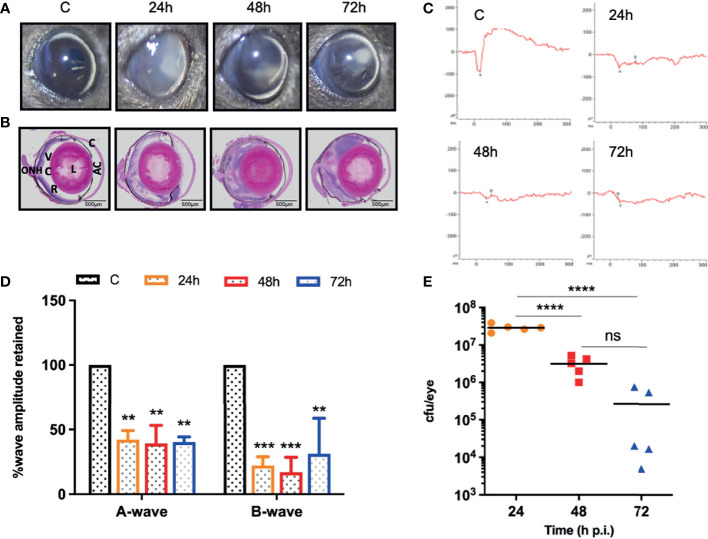
*S. epidermidis* induces retinal tissue damage in the eye. C57BL/6 mice eyes (n = 5 eyes at each time point) were given intravitreal injections of 10^7^ cfu/eye of *S. epidermidis* or PBS (control, C), and eyes were processed at indicated times post-infection. Eyes with PBS injection harvested at 72 h p.i. were used as control. **(A)** Slit-lamp examination was performed, and photomicrographs were taken from representative eyes to visualize corneal haze/opacity. **(B)** At indicated time points post-infection, eyes were enucleated, paraffin fixed, and stained with H&E. **(C)** Scotopic electroretinogram (ERG) analysis was performed to assess retinal function. **(D)** Bar graph showing percent a- and b-wave amplitude retained with respect to control (C) eyes set at 100%. **(E)** At indicated time points, eyes were enucleated and homogenized, and the bacterial burden was estimated *via* serial dilution plating. C, cornea; AC, anterior chamber; L, lens; VC, vitreous chamber; R, retina; ONH, optic nerve head. Statistical analysis was performed using ANOVA with Dunnett’s multiple comparison test **(D)** or Tukey’s multiple comparison test **(E)**, (**) p<0.01 (***) p<0.001(****) p<0.0001; ns, nonsignificant.

Our dose-response study revealed that 10^7^ cfu of *S. epidermidis* causes a significant decline in retinal function and induces persistent inflammation in mouse eyes. Therefore, we decided to use a dose of 10^7^ cfu/eye to determine temporal changes during *S. epidermidis* endophthalmitis *via* assessment of the disease progression up to 72 h. Our data showed that infected eyes had visible opacity and corneal haze at 24 h, which subsequently reduced at 48- and 72-h time points (**Figure 2A**). In contrast, the histological analysis revealed time-dependent retinal tissue damage, with increased retinal folds and heavy cellular infiltrates in the vitreous cavity (**Figure 2B**). This coincided with a significant reduction in retinal function assessed by ERG analysis (**Figure 2C**). The amplitudes of both *a* and *b* waves were significantly decreased at all time points, i.e., 24, 48, and 72 h post-infection (**Figure 2D**). Relatively, the *b* wave amplitudes were lower than *a* wave, indicating impairment of photoreceptor functions. Interestingly, bacterial burden estimation revealed a time-dependent decrease in the viable bacterial count, indicating enhanced bacterial clearance (**Figure 2E**). To ensure whether the observed reduction in bacterial burden is not due to *S. epidermidis* growth defect, we performed an *ex vivo* growth curve using human vitreous. Our data showed that similar to enriched bacterial culture media, *S. epidermidis* exhibited classical log and lag phases of bacterial growth in the vitreous, indicating the unlikelihood of defective bacterial growth (**Figure S1**).

### *S. epidermidis* Induces Inflammatory Responses and PMN Infiltration in the Eye

**Figure 3:**
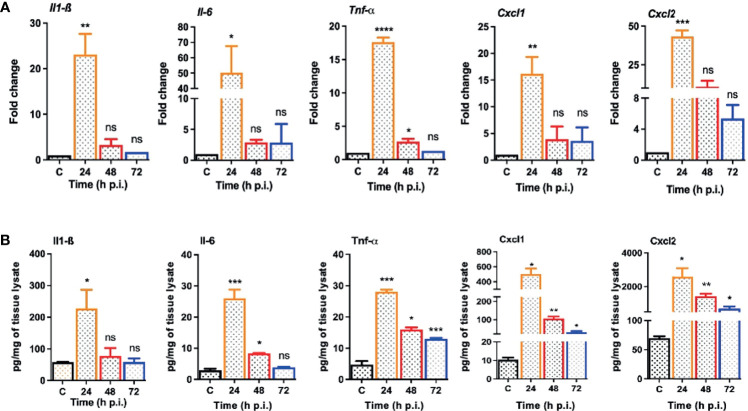
*S. epidermidis* infected eyes exhibit inflammatory response during endophthalmitis. C57BL/6 mice eyes (n = 6 eyes at each time point) were infected by intravitreal injection of 10^7^ cfu/eye of *S. epidermidis* or PBS (control, C) for indicated time points. **(A)** At designated time points, neural retina was harvested and subjected to qPCR analysis for inflammatory mediators, *Il-1β, Il-6*, *Tnf-α*, *Cxcl1*, and *Cxcl2*. **(B)** Whole eye lysates were subjected to ELISA to quantify the protein levels of the same inflammatory mediators. Statistical analysis was performed using ANOVA with Dunnett’s multiple comparison test **(A, B)**, (*) p<0.05 (**) p<0.01 (***) p<0.001 (****) p<0.0001; ns, nonsignificant.

**Figure 4:**
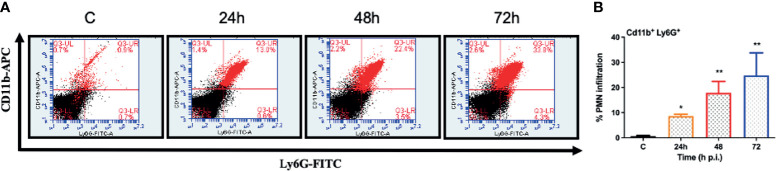
*S. epidermidis* induces PMN infiltration in C57BL/6 mice eyes. C57BL/6 mice eyes (n = 6 eyes) were infected by intravitreal injection of *S. epidermidis* (10^7^ cfu/eye). Eyes with PBS injection harvested at 72 h p.i. were used as control. At indicated time points, retinas were harvested, and single-cell suspensions were stained with anti-CD45-PECy5, anti-CD11b-APC, and anti-Ly6G-FITC antibodies. **(A)** The representative dot plots indicate *S. epidermidis* induced retinal PMN (CD11b-Ly6G double-positive) infiltration. **(B)** The bar graph represents the percentage of neutrophil infiltration at different time intervals with respect to control eyes set at 100%. Statistical analysis was performed using ANOVA with Dunnett’s multiple comparison test **(B)**, (*) p<0.05 (**) p<0.01 ns, nonsignificant.

One of the hallmarks of bacterial infection is the induction of inflammatory response (Kumar et al., 2016; Kumar et al., 2020; Francis et al., 2020). To determine innate immune response during *S. epidermidis* endophthalmitis, we assessed the expression of key inflammatory mediators in infected eyes. Our data showed that *S. epidermidis* induced the expressions of inflammatory cytokines (IL1-β, IL-6, and TNF-α) as well as chemokines (CXCL1 and CXCL2), at both mRNA (**Figure 3A**) and protein (**Figure 3B**) levels. The time-course study revealed that inflammation peaked at 24 h post-infection followed by a significant decline at 48- and 72-h time points. We also noticed that while inflammatory cytokines were drastically reduced at 48 and 72 h, protein levels of the chemokines CXCL1 and CXCL2 were still higher at these time points. Since chemokines play a key role in the recruitment of immune cells, we performed flow cytometry to measure neutrophil infiltration during *S. epidermidis* endophthalmitis. Our data showed a gradual, time-dependent increase in PMN infiltration in *S. epidermidis* infected eyes (**Figure 4A**), as evidenced by increased percentages of CD11b^+^ Ly6G^+^ at all time points (**Figure 4B**). Together, these results indicate the induction of an innate inflammatory response in *S. epidermidis* endophthalmitis.

### *S. epidermidis* Induces TLR Expression and Activation of Inflammatory Signaling

**Figure 5:**
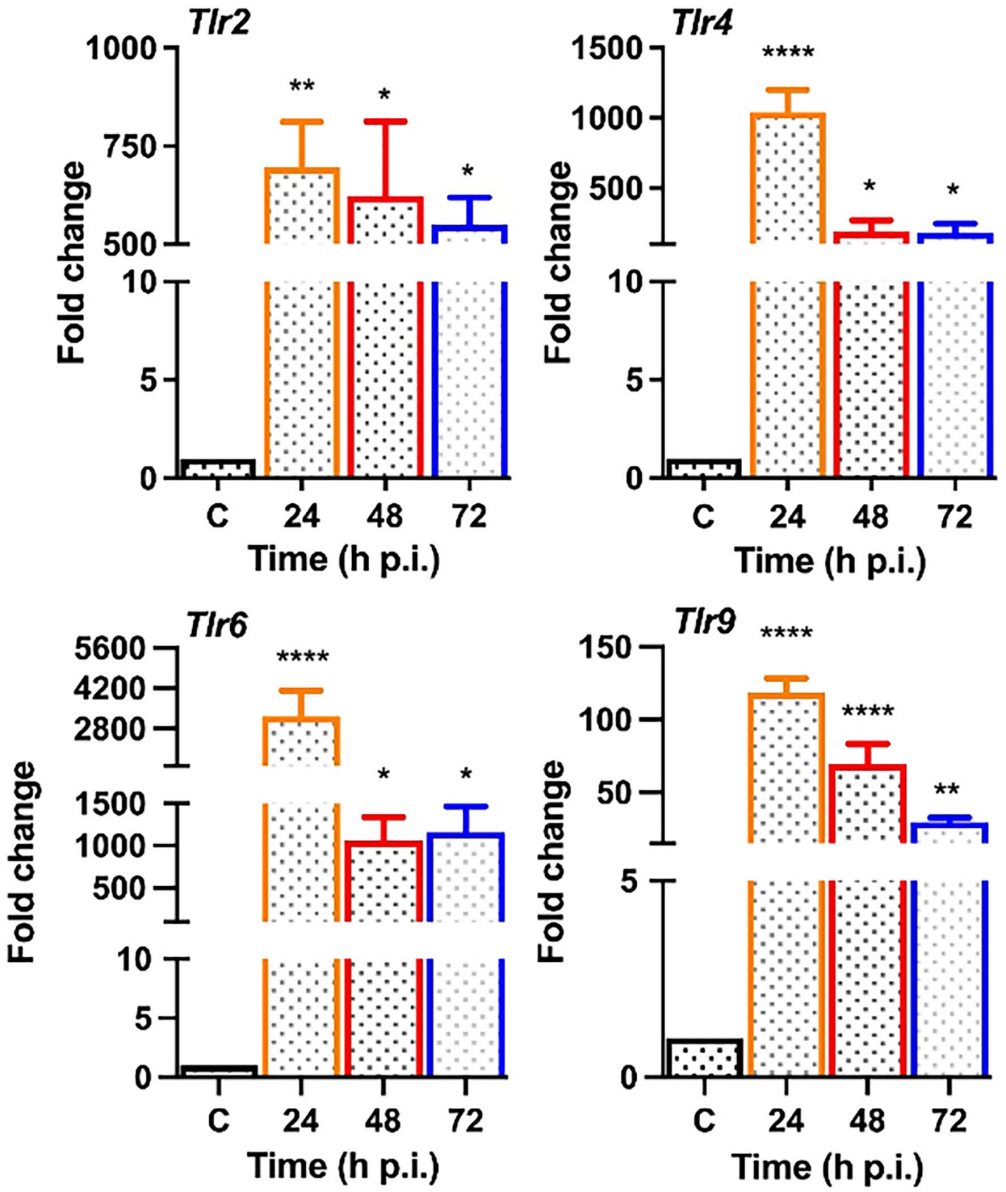
*S. epidermidis* induces Toll-like receptor (TLR) expression in mouse retina. C57BL/6 mice eyes (n = 4 eyes each time point) were infected with *S. epidermidis* (10^7^ cfu/eye) by intravitreal injections, and the retina tissue was harvested at the indicated time points. Eyes with PBS injection harvested at 72 h p.i. were used as control. Infected and control (C) retinal tissues were used for RNA isolation and subjected to qPCR for various Toll-like receptors (*Tlr* 2, 4, 6, 9). The data are presented as fold changes in comparison with uninfected controls. Statistical analysis was performed using ANOVA with Dunnett’s multiple comparison test, (*) p<0.05 (**) p<0.01 (****) p<0.0001.

**Figure 6:**
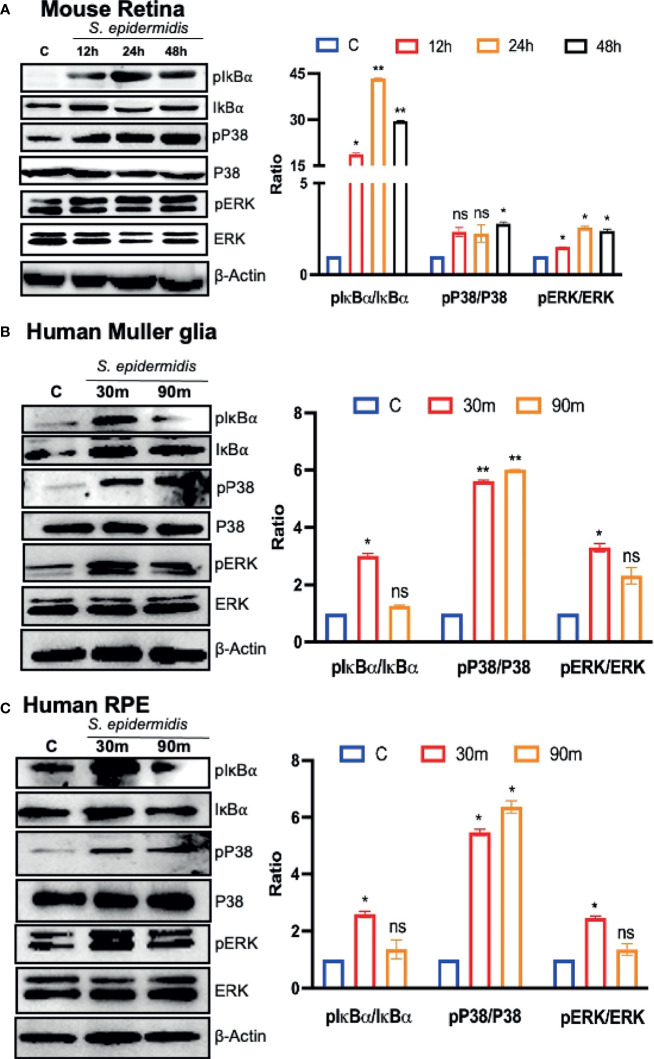
*S. epidermidis* induces inflammatory signaling in mouse retina and cultured human retinal cells. Retinal tissues were harvested from *S. epidermidis* (10^7^ cfu/eye) infected eyes (n = 6 eyes, 2 eyes were pooled into one sample) at the indicated time points. **(A)** The activations of inflammatory signaling proteins were assessed by western blot using specific anti-IκB-α (phospho- and total), anti-ERK (phospho- and total), and anti-P38 (phospho- and total) antibodies, with β-actin as a loading control. In another experiment, cultured human retinal Muller glial cells **(B) (**MIO-M1 cell line) and retinal pigment epithelial cells **(C) (**ARPE-19 cell line) were infected with *S. epidermidis* (MOI of 10) for 30 and 90 min. Cell lysates were probed for indicated phospho- and total proteins. In all experiments, band intensities were quantified by using the Image Studio software and presented as the relative band intensity of phospho/total vs. β-actin. Statistical analysis was performed using ANOVA with Dunnett’s multiple comparison test **(A–C)** (*) p<0.05 (**) p<0.01; ns, nonsignificant.

Upon pathogen invasion, host cells engage TLRs for pathogen recognition and to initiate innate immune responses (Pandey et al., 2013). As our prior studies have implicated the essential role of TLRs in retinal innate defense during endophthalmitis (Kumar et al., 2010; Singh et al., 2014; Talreja et al., 2015), we evaluated their expression in this study. Our data showed that *S. epidermidis* induced the expression of *Tlr2*, *Tlr4*, *Tlr6*, and *Tlr9*, the main TLRs involved in bacterial recognition. Time-course analysis revealed the highest expression of all TLRs at 24 h post-infection with a subsequent decline at 48 and 72 h (**Figure 5**). After pathogen recognition, TLRs activate an inflammatory signaling cascade involving NF-kB and MAPKs (e.g., ERK, P38) resulting in the production of inflammatory mediators. S. *epidermidis* infection resulted in IκB-α phosphorylation, which was detectable and peaked in mouse retinal lysate at 24 h, followed by a slow decline at 48 h. Accompanying the increase in IκB-α phosphorylation, IκB-α degradation was more prominent at 24 h. A similar time-dependent increase was observed in the phosphorylation of P38 and ERK in retinal tissue (**Figure 6A**). We also assessed the activation of these pathways in retinal Muller glia and RPE, which were shown to play roles in retinal innate defense during intraocular infection (Lenkowski and Raymond, 2014; Singh et al., 2014; Singh et al., 2017). Our data showed that *S. epidermidis* induced the activation of NF-kB, P38, and ERK signaling in Muller glia (**Figure 6B**) and RPE (**Figure 6C**) as evidenced by increased phosphorylation at both 30- and 90-min time points. Altogether, these results indicate that both retina and retinal cells directly respond to *S. epidermidis* and activate TLR-mediated inflammatory signaling pathways.

### *S. epidermidis* Induces the Production of Inflammatory Mediators in Retinal Cells

**Figure 7:**
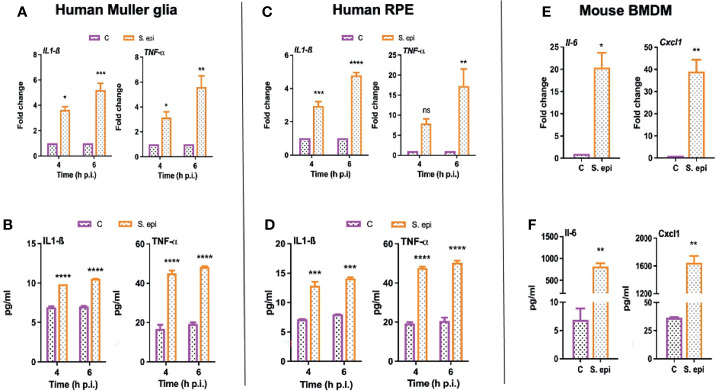
*S. epidermidis* induces inflammatory responses in cultured mouse BMDMs and human retinal cells. **(A, B)** Human retinal Muller glial cells (MIO-M1 cell line) **(C, D)** human retinal pigment epithelial cells (ARPE-19 cells), and **(E, F)** mouse bone marrow-derived macrophages (BMDMs) were infected with *S. epidermidis* (MOI of 10) for 4 or 6 h. At indicated time points, cells were harvested for qPCR analysis of inflammatory cytokines/chemokines **(A, C, E)**, and culture supernatants were used for ELISA to quantify their protein levels **(B, D, F)**. Statistical analysis was performed using ANOVA with Sidak’s multiple comparison test **(A–D)** and t-test **(E, F)** (*) p<0.05 (**) p<0.01 (***) p<0.001 (****) p<0.0001.

Next, we assessed the biological relevance of the induced signaling pathways; we determined the effect of *S. epidermidis* on the expression and secretion of inflammatory cytokines. We observed that human Muller glia challenged with *S. epidermidis* had increased expression of IL-1β and TNF-α both at the mRNA (**Figure 7A**) and protein (**Figure 7B**) levels. Moreover, the response was found to be time-dependent with a relative increase at the 6-h time point. Human RPE cells also exhibited a similar pattern of IL-1β and TNF-α mRNA (**Figure 7C**) and protein (**Figure 7D**) expression in response to *S. epidermidis* infection. PMNs and monocytes/macrophages are also major infiltrating innate immune cells during ocular infections (Singh et al., 2021); therefore, we decided to assess the inflammatory response of mouse BMDMs. To this end, our data showed that *S. epidermidis* induced robust *Il-6* and *Cxcl1* mRNA (**Figure 7E**) and protein expressions (**Figure 7F**) in cultured BMDMs. The induced expression of other inflammatory cytokines such as *Il-1β* and *Tnf-α* both at mRNA and proteins was also detected in BMDMs **(** **Figure S2** **)**. It should be noted that there was a differential response based on cell types, with BMDMs expressing much higher levels of inflammatory mediators compared to Muller glia and RPE. These results suggest that retinal cells possess the ability to respond to *S. epidermidis* infection by secreting inflammatory mediators.

## Discussion

**Figure 8:**
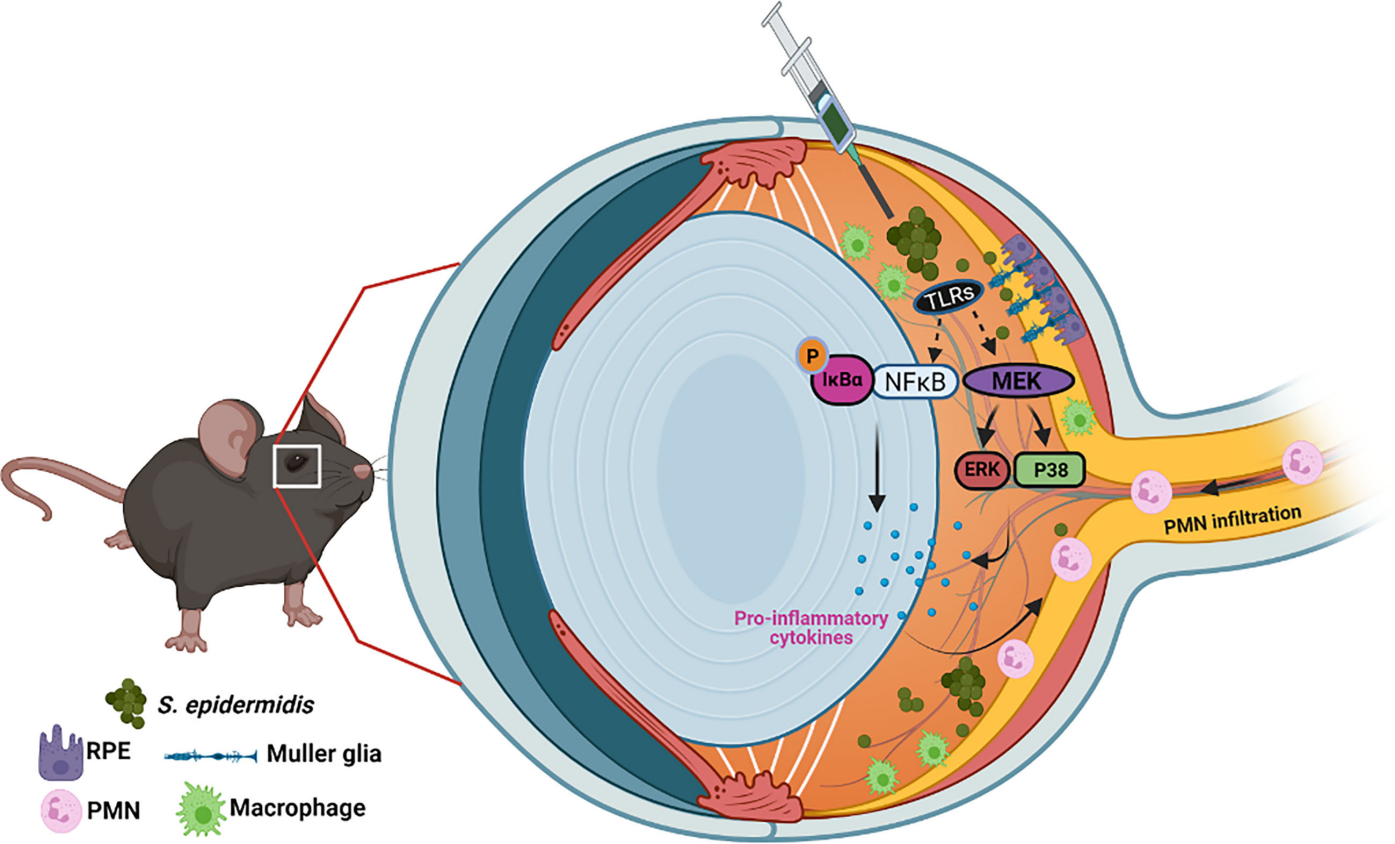
Schematic of pathobiology of *S. epidermidis* endophthalmitis. Intraocular inoculation of *S. epidermidis* is recognized by TLRs expressed on infiltrating immune cells such as monocytes/macrophages, retinal residential cells, Muller glia, and RPE. Upon activation of inflammatory signaling, these cells secrete inflammatory cytokine and chemokines to recruit PMNs. Although essential to control bacterial growth, increased inflammation and PMN infiltration cause collateral retinal tissue damage during endophthalmitis, culminating in reduced visual function and blindness.

*S. epidermidis* and other coagulase-negative *Staphylococci* (CoNS) are the most commonly recovered bacterial species from endophthalmitis patients (Miller et al., 2019; Malmin et al., 2021). They are also key constituents of the human microflora and usually colonizing moist areas, thereby considered as opportunistic pathogens (Kloos and Musselwhite, 1975; Grice et al., 2009). Today, CoNS represent one of the major nosocomial pathogens impacting human health (Rogers et al., 2009; Becker et al., 2014; Nguyen et al., 2017). In the eye, *S. epidermidis* colonizes the conjunctiva (Schleifer and Kloos, 1975). Therefore, during ocular surgeries, *S. epidermidis* can enter inside the eye through contaminated surgical devices and result in endophthalmitis (Lowy, 1998; Durand, 2013). Although most clinical studies have reported *S. epidermidis* to cause less severe endophthalmitis as compared to *S. aureus* (Pichi et al., 2014), few experimental studies have been conducted to understand its pathobiology (Pleyer et al., 1992; Ravindranath et al., 1995; Ravindranath et al., 1997). Because the eye is an immune privilege organ, it could provide a conducive milieu for opportunistic pathogens. Moreover, the treatment of CoNS infections is becoming more challenging due to the emergence of multidrug resistance among large proportions of ocular isolates (Asbell et al., 2015; Asbell et al., 2020). Therefore, it is crucial to understand the host–pathogen interactions during ocular CoNS infections. In this study, using *in vitro* and *in vivo* experimental models, we provide mechanistic insights into the pathogenesis of *S. epidermidis* endophthalmitis (**Figure 8**). Most importantly, our study shows that while the bacterial burden gradually declines in the infected eye, neutrophil infiltration and retinal tissue damage continue until 72 h, resulting in significant vision loss. Collectively, to the best of our knowledge, this is the first study to elucidate the innate immune responses and pathobiology of *S. epidermidis* endophthalmitis in a murine model.

In healthy individuals, *S. epidermidis* is considered a low virulence pathogen as compared to *S. aureus* (Pichi et al., 2014). However, in immunocompromised individuals, it possesses the greatest pathogenic potential among CoNS (Giese and Mondino, 2001; Paharik and Horswill, 2016). Although *S. epidermidis* pathogenesis has been extensively studied in the context of foreign body infections (Uçkay et al., 2009), specifically the biofilm formation, little is known about its virulence factors responsible for causing ocular infections. Thus, to establish endophthalmitis, we performed a dose-response study by intravitreal injections of *S. epidermidis*. We found that bacterial inoculum in the range of 10^5^ to 10^7^ cfu/eye induced an inflammatory response and opacity within 24 h with 10^7^ cfu/eye dose causing increased disease severity. This observation indicates that unlike *S. aureus*, which induces endophthalmitis at 5,000 cfu/eye (Talreja et al., 2015), *S. epidermidis* needed a much higher inoculum. This is also consistent with prior studies in rat and rabbit models, where authors reported that a certain threshold of inoculum was needed to overcome immune responses and for persistent infection (Pleyer et al., 1992; Maxwell et al., 1993; Ravindranath et al., 1995). However, when we performed a time-dependent study using 10^7^ cfu/eye dose of *S. epidermidis*, we found that bacterial burden was only slightly increased at 24-h time followed by a rapid decline at later time points. In our prior studies with *S. aureus*, we showed that bacteria continue to grow inside the eye from 24 to 72 h (Shamsuddin and Kumar, 2011; Kumar et al., 2016; Singh et al., 2021); therefore, we used the same timeline. However, future studies, with the early time points (6 and 12 h) might be helpful to dissect intraocular proliferation of *S. epidermidis.* In *ex vivo*, we found that *S. epidermidis* grew in the human vitreous up to 24 h; however, the growth rate was lower than enriched bacterial growth media. The observed differences are likely due to lesser availability of nutrients in the vitreous rather than defect in bacteria growth.

The gross eye exam revealed the clearance of anterior chamber opacity at 48 and 72 h, indicating a recovery. These results suggest that mouse eye/vitreous can kill *S. epidermidis*, a phenomenon known as spontaneous sterilization discovered by Meredith et al. in a rabbit model (Meredith et al., 1990). However, our use of a mouse model provides the added advantage of versatility, which is needed to properly study the pathogenesis of *S. epidermidis* endophthalmitis *via* the wide availability of genetic and immunological tools. Similar to our prior study in *S. aureus* endophthalmitis (Singh et al., 2020), we did not observe any significant difference in the pathogenesis of *S. epidermidis* endophthalmitis in male *versus* female mice, indicating the applicability of our findings across genders.

Once inside the vitreous, retinal cells recognize pathogens *via* pattern recognition receptors such as TLRs (Singh and Kumar, 2015) and elicit inflammatory signaling pathways (Kumar and Shamsuddin, 2012; Pandey et al., 2013). Our data show that intravitreal inoculation of *S. epidermidis* initiated retinal innate immune response by inducing the expression of several TLRs, followed by induction of NFκB and MAPK signaling with increased phosphorylation of IκBα, ERK, and P38 proteins in mouse retinal tissue. Because the retina is a complex tissue, to determine the contribution of retinal cell types in innate response to *S. epidermidis*, *in vitro* studies were performed using retinal Muller glia and RPE, representing retinal residential cells (Strauss, 2005; Kumar et al., 2013; Linehan and Fitzgerald, 2015). We found that both Muller glia and RPE exhibited the activation of these signaling pathways within 30 min of *S. epidermidis* exposure, indicating their role in orchestrating an innate response during endophthalmitis. We previously reported that retinal cells respond to bacterial virulence factors *via* TLRs (Shamsuddin and Kumar, 2011; Kumar and Shamsuddin, 2012; Kumar et al., 2013; Singh et al., 2014). Moreover, Muller glia cells were found to participate in clearance of *S. aureus* by secreting antimicrobial peptides (Shamsuddin and Kumar, 2011) and the production of both ROS and NO, and by phagocytosis (Singh et al., 2014). Hence, we speculate similar antimicrobial mechanisms exhibited by Muller glia during *S. epidermidis* endophthalmitis.

The eye is protected from systemic circulation due to the presence of blood-retinal barrier (BRB), which is essential in maintaining visual function. It is composed of two major cell types: the endothelial cell, which constitutes inner BRB, and a single layer of RPE cells forming the outer barriers (Singh et al., 2019). Both these cells play a critical role in controlling the infiltration of immune cells during retinal infection, including endophthalmitis (Coburn et al., 2016). Because of their intimate contact with choroid and other retinal cell types (e.g., photoreceptors), RPE senses the presence of pathogenic stimuli coming from both choroid as well as from vitreous. Thus, RPE plays a pivotal role in eliciting innate immune responses *via* activation of microbial sensors (TLRs, NOD-like receptors, NLR), cytokine/chemokine production, and an array of complement components to combat retinal infections (Detrick and Hooks, 2010). Inside the retina, the activated RPE cells interact with microglia, thereby acting as an inflammatory signal to upregulate microglial cells to secrete cytokines (Kumar et al., 2004). Furthermore, RPE cells constitutively express AMPs, and its expression is regulated during pathogen insults to protect the blood-retinal barrier (Liu et al., 2021). Studies have also shown that iNOS triggered by secreted cytokines in RPE against *Staphylococcus* infections mediates anti-inflammatory effects that, in turn, prevent IDO-1-dependent tissue damage (Spekker-Bosker et al., 2019). Our data show the activation of inflammatory signaling and the production of inflammatory mediators by RPE in response to *S. epidermidis* infection indicate their role in the activation of an innate immune response.

Neutrophils are the primary infiltrating cells of the vitreous during endophthalmitis (Miller et al., 2019; Kumar et al., 2020; Singh et al., 2021). Although neutrophil deficiency reduces ocular inflammation, it comes with the price of increased bacterial proliferation in the eye (Talreja et al., 2014; Talreja et al., 2015). Thus, a fine balance is needed to have sufficient PMN infiltration to kill pathogens without causing collateral ocular tissue damage. Our data show that *S. epidermidis* burden is decreased in infected eyes as time progresses coinciding with increased PMN infiltration. Thus, PMNs are likely to be responsible for enhanced bacterial clearance of *S. epidermidis* in later stages of infection. As neutrophils are the first responder during infection, we found that their depletion exacerbates bacterial and fungal endophthalmitis (Talreja et al., 2014; Gupta et al., 2019), indicating an indispensable role. We anticipate that in neutropenic mice, *S. epidermidis* might grow more; however, whether inflammation or disease outcome be better or worse needs further investigation.

Our *in vitro* study using *S. epidermidis* infected mouse BMDMs also showed secreted inflammatory cytokines and chemokines. Thus, both PMNs and monocyte/macrophages, while eradicating bacteria, contribute to ocular inflammation. The influx of these phagocytic cells into the eye is facilitated due to BRB breakdown and the production of inflammatory mediators in endophthalmitis. We found that the levels of IL-6, TNF-α, and IL-1β cytokines peaked at 24 h followed by a steep decline. However, the decrease in protein levels of chemokines, CXCL1, and CXCL2 was more gradual. We believe that these chemokines might be responsible for continued infiltration of PMNs. In addition to chemokines, induced expression of E-selectin and ICAM-1 cells has been implicated in the infiltration of PMNs during endophthalmitis (Giese et al., 2000). The expression of these adhesion molecules is predominantly observed on endothelial cells in the iris, ciliary body, and choroid, indicating that these are the main sites for cellular infiltration into the eye in endophthalmitis. We observed that while bacterial burden and inflammatory cytokines subsided in *S. epidermidis* endophthalmitis, histological analysis revealed significant damage to the retina culminating in a reduced ERG response. These findings have clinical implications in endophthalmitis caused by typically low virulence pathogen because the clarity of vitreous is often used as an endpoint in laboratory diagnosis to terminate antimicrobial therapy. However, our study indicates that inflammation persists despite declining bacterial numbers. Similarly, in bacterial meningitis, antibiotic treatment has been shown to increase inflammation and tissue destruction due to the release of bacterial cell wall components (Quagliarello and Scheld, 1992). Thus, we propose to use adjunct anti-inflammatory therapeutics with antibiotics for the treatment of bacterial endophthalmitis (Francis et al., 2020; Singh et al., 2021).

In conclusion, our findings suggest that although *S. epidermidis* was able to establish endophthalmitis in mice at a relatively higher inoculum, it elicited significant inflammation and mediated retinal tissue damage in the eye. Further studies are needed to determine precise mechanisms underlying increased PMN infiltration to better understand the pathobiology of *S. epidermidis* endophthalmitis, which would aid in the development of therapeutic modalities.

## Material and Methods

### Bacterial Strain and Growth Conditions

The bacterial strain used in this study is a coagulase-negative strain, *S. epidermidis*, identifiable as ATCC12228. The strain was routinely cultured in tryptic soy medium (TSA or TSB; Sigma, St. Louis, MO) at 37°C for all the experiments. For *in vitro* and *in vivo* infection experiments, overnight-grown bacteria were rinsed and diluted in 1X PBS to achieve the desired colony forming units (cfu). For *S. epidermidis* growth curve analysis, 180 µl of homogenized human vitreous, Muller Hinton broth (MHB), or TSB was added to each well of a microtiter plate and 20 µl of bacterial suspension (5x10^4^ cfu/ml). After 6, 12, 24, and 48 h from each well, 20-µl aliquots were obtained and serially diluted to determine the bacterial count on tryptic soy agar (TSA).

### Retinal Cell Culture and Maintenance

The human retinal pigment epithelial cell line ARPE-19 was maintained in Dulbecco’s modified Eagle’s medium nutrient mixture F-12 (DMEM F-12), whereas the human retinal Muller glia cell line MIO-M1 was cultured in DMEM GlutaMAX, both with supplementation of 10% fetal bovine serum (FBS) and 1% penicillin-streptomycin antibiotic solution at 37°C in 5% CO_2_. However, prior to infection, the cells were cultured in serum and antibiotic-free media and infected with *S. epidermidis* (MOI, 10:1) for various time points. The cells were used for extraction of RNA or proteins, whereas cell-free culture media were collected for cytokine ELISA.

### Isolation of Bone Marrow-Derived Macrophages

The isolation of bone marrow-derived macrophages (BMDMs) was done from C57BL/6 mice as described earlier (Singh et al., 2021). Briefly, 6–8 weeks old mice were euthanized, and bone marrows were extracted from their tibias and femurs with RPMI-1640 media containing 10% FBS and 0.2 mM EDTA, maintaining an aseptic condition inside a BSL-2 cabinet. Cells were centrifuged at 400 x g for 5 min at 4°C, followed by the lysis of RBCs in differential NaCl solutions. The cells were then rinsed with the media and transferred to a Petri dish containing an RPMI-1640 medium supplemented with 10% FBS, 1% antibiotic, and 10 ng/ml macrophage colony-stimulating factor (M-CSF) to allow macrophage differentiation. Cells were maintained in a 5% CO_2_ incubator at 37°C. After 6 days post isolation, BMDMs were seeded in six-well plates and challenged with *S. epidermidis* (MOI, 10:1). The cells were used for the extraction of RNA or proteins, whereas culture media were collected for cytokine ELISA.

### Cytokine ELISA

After infection, the culture supernatants from *in vitro* experiments were collected, and the levels of IL-1β, IL-6, TNFα, CXCL1, and CXCL2 were determined by ELISA using commercially available kits as described previously (Talreja et al., 2015). ELISA was performed as per the manufacturer’s instructions (R&D systems, Minneapolis, MN). For *in vivo* cytokine estimation, the whole eyes were enucleated, homogenized in 1X PBS by beating against stainless-steel beads in a Tissue lyser (Qiagen, Valencia, CA, USA), and centrifuged, and the lysates were subjected to ELISA as mentioned above. It is pertinent to note that, before performing ELISA, protein estimation was done using the BCA method to ensure that equal protein concentrations were used for each sample.

### RNA Extraction, cDNA Synthesis, and qPCR

Total RNA was extracted from cultured cells or mouse retina using a TRIzol reagent as per the manufacturer’s protocol (Invitrogen, Carlsbad, CA). Next, cDNA was synthesized using 1 μg of the isolated RNA using a Maxima first-strand cDNA synthesis kit according to the manufacturer’s instructions (Thermo Scientific, Rockford, IL). The cDNA was then subjected to qRT-PCR on a StepOnePlus Real-Time PCR System (Applied Biosystems, Foster City, CA, USA) using gene-specific PCR primers synthesized from Integrated DNA Technologies (Coralville, IA, USA) with a PCR condition of initial denaturation at 94°C for 5 min, followed by 40 cycles of denaturation (94°C, 45 s), annealing (60°C, 1 min), and extension (72°C, 45 s), with a final extension at 72°C for 10 min. The data were analyzed as a comparative ΔΔC_T_ method and were presented corresponding to the fold-change differences in gene expression in test samples with respect to control.

### Animal Housing and Use

Both male and female C57BL/6 (B6) mice (age, 6–8 weeks) were purchased from the Jackson Laboratory (Bar Harbor, ME, USA) and were housed in a restricted access DLAR facility at the Kresge Eye Institute, maintained in a 12:12 light/dark cycle, and fed with LabDiet rodent chow (Labdiet; Pico Laboratory, St. Louis, MO, USA) and water *ad libitum*. Both male and female mice, around 8 weeks of age, were used. Mice were treated in compliance with the Association for Research in Vision and Ophthalmology (ARVO) Statement for the Use of Animals in Ophthalmic and Vision Research, and all procedures were approved by the Institutional Animal Care and Use Committee (IACUC) of Wayne State University under protocol # IACUC-19-03-1012.

### Patient Vitreous Collection

Human vitreous samples were collected under sterile conditions from patients undergoing vitrectomy and had signed a preoperative informed consent to use the excised vitreous fluid for basic and clinical research. The protocol and study design were approved by the Wayne State University School of Medicine Institutional Review Board. Collected samples were stored in -80°C until further use.

### Induction of Bacterial Endophthalmitis

Bacterial endophthalmitis was induced in B6 mice by intravitreal injection with specified doses of *S. epidermidis*. As per our IACUC approved protocol, only the eye of each mouse can be injected with either sterile PBS (serving as control) or bacteria. To obtain a 10^7^ inoculum, we concentrated 10 ml of bacterial culture equivalent to 1 O.D (~10^8^ cfu/ml) by pelleting and resuspending in 200 μl of PBS. Mice were anesthetized with ketamine and xylazine, and intravitreal injections of PBS or bacteria (2-μl volume) were performed using a 34-gauge needle under a microscope. This procedure is routinely performed in the lab and reported in our several studies (Kumar et al., 2010; Talreja et al., 2014; Kumar et al., 2016; Singh et al., 2020; Singh et al., 2021). Disease progression was monitored using slit-lamp examination and testing retinal function using electroretinogram (ERG). Following the desired time point post-infection, enucleated eyes were subjected to bacterial burden estimation, cytokine/chemokine ELISA, polymorphonuclear neutrophil (PMN) infiltration, and histopathology, as described in the following sections.

### Bacterial Burden Estimation

Bacterial densities in infected eyes of WT mice were assessed using the standard serial dilution and the bacterial plate count method. At the indicated time points, the eyes were enucleated and homogenized in sterile 1X PBS in a tissue lyser (Qiagen, Valencia, CA, USA), followed by serial dilution and plating on tryptic soy agar (TSA) plates. Results were expressed as mean ± SD number of colony-forming units (cfu)/eye.

### PMN Infiltration

Flow cytometry was performed to estimate the infiltration of neutrophils in infected eyes as described earlier (Talreja et al., 2015). In brief, the retinas from euthanized mice were isolated and digested with Accumax (Millipore) for 10 min at 37°C, with intermittent mixing using a 22-gauge needle and a syringe. Next, to obtain a single-cell suspension, the lysate was filtered through a 40-μm cell strainer (BD Falcon, San Jose, CA, USA). The cells were then incubated with Fc Block (BD Biosciences) for 30 min, followed by three times washing with PBS containing 0.5% bovine serum albumin (BSA). For staining the cells, phycoerythrin (PE)-Cy5-conjugated CD45, Ly6G-FITC, and CD11b-APC antibodies (BD Biosciences) were used to incubate the cells for 30 min in the dark. Following incubation, cells were washed and suspended in sheath fluid. The stained cells were acquired on the Accuri C6 flow cytometer (BD Biosciences) at the NEI P30 immunology core at the Kresge Eye Institute. Data were analyzed using the manufacturer’s software.

### Retinal Function Testing

Scotopic electroretinography (ERG) was done to evaluate retinal function in *S. epidermidis* induced endophthalmitis as described previously (Francis et al., 2020). Briefly, following overnight dark adaptation, ERGs were recorded in control and infected mice eyes using the Celeris ERG system (Diagnosis LLC, Lowell, MA, USA) according to the manufacturer’s instructions. The ERG a-wave was measured as an amplitude between the ERG baseline and the first negative peak, and the ERG b-wave was measured as an amplitude between the first negative peak and the first positive peak. Data were analyzed with respect to placebo control eyes.

### Ocular Histology

Mice were euthanized and eyes were enucleated, fixed in 4% formalin for histopathological analysis. The embedding, sectioning, and hematoxylin and eosin (H&E) staining of the tissues were done by Excalibur Pathology, Inc. (Oklahoma City, OK, USA). The slides were further scanned under the PathScan Enabler IV (Meyer Instruments, Inc., Houston, TX, USA) to obtain images.

### Western Blotting

Following infection, proteins were extracted from cultured cells after being washed with 1X PBS and lysed with radioimmunoprecipitation assay (RIPA) buffer, supplemented with protease and phosphatase inhibitor cocktails. Retinal tissues from two eyes were pooled in RIPA buffer and sonicated, and lysates were obtained after centrifugation. Total protein concentration was detected using a Micro BCA protein assay kit (Thermo Scientific, Rockford, IL). For western blot, the samples were run on SDS polyacrylamide gels and electrotransferred to 0.45-μm nitrocellulose membranes using a wet blot transfer. The membranes were then treated with 5% skim milk in TBST (20 mM Tris HCl [pH 7.6], 0.15 M sodium chloride, and 0.5% Tween 20) for 1 h at RT and further incubated with respective primary antibodies (Cell Signaling Technology, USA or Santa Cruz Biotechnology, USA) as per the manufacturer’s protocol for overnight on a rocker at 4°C. After washing thrice with TBST, the membranes were further treated with horseradish peroxidase (HRP)-conjugated appropriate secondary antibodies (anti-mouse or anti-rabbit Ig) for 2 h. Following three TBST washes, the blots were developed with a Super Signal West Femto chemiluminescent substrate kit (Thermo Scientific, Rockford, IL). To generate quantitative data, immunodetected protein band intensities were measured using the Image Studio software (LI-COR Biosciences, NE, USA).

### Statistical Analysis

All the assays were performed independently three times in biological triplicates, and graphs were plotted showing mean ± standard deviation. The data were analyzed using either Student’s t-tests or ANOVA with the help of GraphPad Prism version 8.1 (Graph Pad, CA, USA). A confidence interval of 95% was maintained for all experimental values. A p-value < 0.05 was considered statistically significant.

## Data Availability Statement

The original contributions presented in the study are included in the article/**Supplementary Material**. Further inquiries can be directed to the corresponding author.

## Ethics Statement

The animal study was reviewed and approved by the Institutional Animal Care and Use Committee (IACUC) of Wayne State University under protocol # IACUC-19-03-1012.

## Author Contributions

SD, SS, and AK conceived the project and designed the experiments. SD and SS performed experiments and analyzed the data. AK contributed reagents/materials/analysis tools. SD and AK wrote the manuscript. All authors contributed to the article and approved the submitted version.

## Funding

This study was supported by NIH grants R01EY026964 and R01EY027381. Our research is also supported in part by an unrestricted grant from Research to Prevent Blindness (RPB) to the Kresge Eye Institute/Department of Ophthalmology, Visual, and Anatomical Sciences. The immunology core is supported by an NEI center grant P30EY004068.

## Conflict of Interest

The authors declare that the research was conducted in the absence of any commercial or financial relationships that could be construed as a potential conflict of interest.

## Publisher’s Note

All claims expressed in this article are solely those of the authors and do not necessarily represent those of their affiliated organizations, or those of the publisher, the editors and the reviewers. Any product that may be evaluated in this article, or claim that may be made by its manufacturer, is not guaranteed or endorsed by the publisher.
