# Supplementary material for: Bacterial Burden Declines But Neutrophil Infiltration and Ocular Tissue Damage Persist in Experimental Staphylococcus epidermidis Endophthalmitis
Source: Front Cell Infect Microbiol. 2021 Nov 17;11:780648. doi: 10.3389/fcimb.2021.780648 (PMC8635919; doi:10.3389/fcimb.2021.780648)
Supplement: Supplementary file 1 [file DataSheet_1.docx]

**Supplementary data**

**
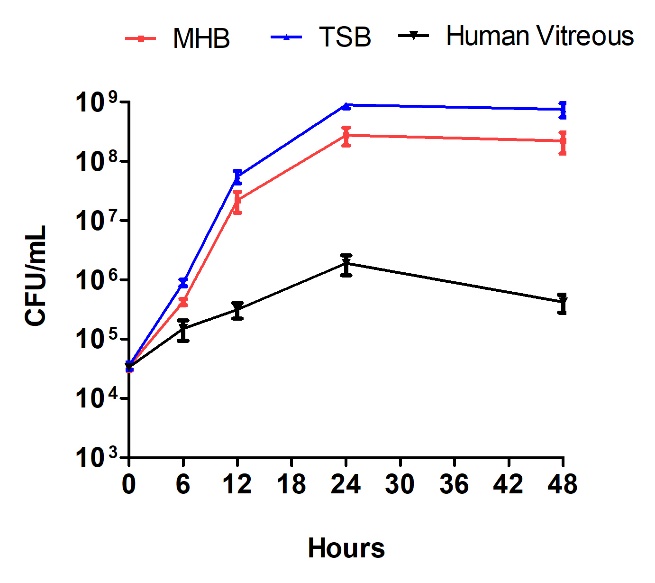
**

**Fig. S1:** *In vitro* growth curves of analysis of the S. epidermidis in Muller Hinton broth (MHB), Tryptic soya broth (TSB), or human vitreous. The data represented is cumulative of two independent experiments performed in triplicates for the each time point.


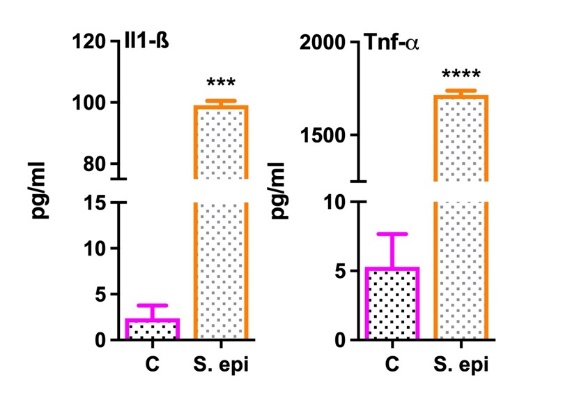


**B**


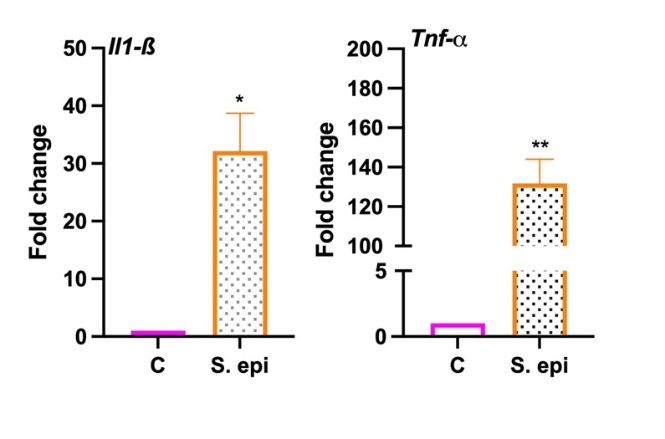


**A**

**Fig. S2:** Mouse bone marrow-derived macrophages (BMDMs) were infected with S. epidermidis (MOI of 10) for 6h. Cells (n=3) were then harvested for qPCR **(A)** and cell-free culture supernatants were used for ELISA **(B)** assays of inflammatory cytokines. Statistical analysis was performed using t-test (A-B) (*) p<0.05 (**) p<0.01 (***) p<0.001 (****) p<0.0001.
